# Comparison of pathogenicity of subtype H9 avian influenza wild-type viruses from a wide geographic origin expressing mono-, di-, or tri-basic hemagglutinin cleavage sites

**DOI:** 10.1186/s13567-020-00771-3

**Published:** 2020-03-31

**Authors:** Rokshana Parvin, Jan Schinkoethe, Christian Grund, Reiner Ulrich, Franziska Bönte, Klaus P. Behr, Matthias Voss, Mohammed A. Samad, Kareem E. Hassan, Christine Luttermann, Martin Beer, Timm Harder

**Affiliations:** 1 Institute of Diagnostic Virology, Federal Research Institute for Animal Health, Friedrich-Loeffler-Institute (FLI), Suedufer 10, 17493 Greifswald-Insel Riems, Germany; 2 Department of Pathology, Faculty of Veterinary Science, Bangladesh Agricultural University, Mymensingh, 2202 Bangladesh; 3 Institute of Veterinary Pathology, Faculty of Veterinary Medicine, University of Leipzig, An den Tierkliniken 33, 04103 Leipzig, Germany; 4 University of Applied Sciences Wedel, Feldstraße 143, 22880 Wedel, Germany; 5 AniCon Labor GmbH, Mühlenstraße, 49685 Höltinghausen, Germany; 6 Lohmann Tierzucht GmbH, Veterinär-Labor, Abschnede 64, 27472 Cuxhaven, Germany; 7 NRL-AI, Bangladesh Livestock Research Institute (BLRI), Savar, Dhaka, Bangladesh; 8 Poultry Diseases Department, Faculty of Veterinary Medicine, Beni-Suef University, Beni Suef, Egypt; 9 Institute of Immunology, Friedrich-Loeffler-Institute, Greifswald-Riems, Germany

## Abstract

An intravenous pathogenicity index (IVPI) of > 1.2 in chickens or, in case of subtypes H5 and H7, expression of a polybasic hemagglutinin cleavage site (HACS), signals high pathogenicity (HP). Viruses of the H9N2-G1 lineage, which spread across Asia and Africa, are classified to be of low pathogenicity although, in the field, they became associated with severe clinical signs and epizootics in chickens. Here we report on a pre-eminent trait of recent H9N2-G1 isolates from Bangladesh and India, which express a tribasic HACS (motif PAKSKR-GLF; reminiscent of an HPAIV-like polybasic HACS) and compare their features to H9Nx viruses with di- and monobasic HACS from other phylogenetic and geographic origins. In an in vitro assay, the tribasic HACS of H9N2 was processed by furin-like proteases similar to bona fide H5 HPAIV while some dibasic sites showed increased cleavability but monobasic HACS none. Yet, all viruses remained trypsin-dependent in cell culture. In ovo, only tribasic H9N2 viruses were found to replicate in a grossly extended spectrum of embryonic organs. In contrast to all subtype H5/H7 HPAI viruses, tribasic H9N2 viruses did not replicate in endothelial cells either in the chorio-allantoic membrane or in other embryonic tissues. By IVPI, all H9Nx isolates proved to be of low pathogenicity. Pathogenicity assessment of tribasic H9N2-G1 viruses remains problematic. It cannot be excluded that the formation of a third basic amino acid in the HACS forms an intermediate step towards a gain in pathogenicity. Continued observation of the evolution of these viruses in the field is recommended.

## Introduction

Infections with low pathogenicity avian influenza viruses (LPAIV) of subtype H9N2 remain highly prevalent in poultry in many countries in Asia, the Middle East, and Africa [1]. Since the mid-1990s, Asian H9N2 viruses attracted particular attention due to their panzootic properties and diverging evolution into several major lineages [2, 3]. Descendants of the prototype strain A/Quail/Hong Kong/G1/97, the G1 lineage, donated, by reassortment, genome segments to several co-circulating highly pathogenic avian influenza viruses (HPAIV) of subtypes H5N1 [4, 5], H5N6 [6], LPAIV H7N3 [7], zoonotic H10N8 [8], and the highly zoonotic Chinese H7N9 lineage [9]. A Q226L (H3 numbering) substitution in the receptor binding unit of the H9-G1 hemagglutinin (HA) protein skewed binding specificity towards human-like alpha 2,6 sialic acid receptors [10]. Consequently, these mutants have caused several human cases of H9N2 infection: 26 in China [11], three in Bangladesh [12] and three in Egypt [13]. Independent of the lineage, all H9 strains are considered to be of low pathogenicity based on the lack of inducing mortality in the standardized in vivo pathotyping tests in chickens [14]. Despite their classification as LPAIV, they are associated with severe disease in poultry leading to significant economic losses [15, 16]. It has been suggested that the strikingly distinct clinical signs induced in experimental and natural H9N2 infections are due to opportunistic viral and bacterial co-infections with H9N2 acting in synergy as an immunosuppressive agent [16–19].

The influenza HA glycoprotein is an important but not the sole determinant of influenza virus pathogenicity [20]. HA properties affect host as well as tissue tropism [21]. Regulated endoproteolytical cleavage by appropriate host-derived proteases of the HA precursor protein (HA0) into disulfide-linked HA1 and HA2 subunits is crucial to ensure infectivity of progeny virions [22]. The HA endoprotease motif (referred to as the HA cleavage site, HACS) is strongly correlated with pathogenicity for the H5 and H7 subtype viruses in avian hosts. HA harboring a monobasic cleavage site [R*GLF] is processed by trypsin-like serine proteases [23] restricting infection to the enteric or respiratory epithelia of birds where such proteases are expressed. Mild, if any, clinical signs usually result from experimental infection of chickens with such viruses. In contrast, viruses that exhibit a polybasic cleavage site motif [R-X-K/R-R*GLF] allow endoproteolytic activation in the trans-Golgi network by ubiquitous members of the proprotein convertase family such as furin [24, 25]. Processability of viral HA by ubiquitous proteases facilitates systemic virus spread and predisposes to lethal disease [26]. There is ample evidence that conversion by spontaneous mutation of a monobasic cleavage site into a polybasic one is at the basis of the pathogenicity shift from LP to HP phenotype in subtypes H5 and H7 [23]. Why and when such conversion events occur remains essentially unclear [27].

Until very recently, all reported H9 HA sequences encoded a mono- or dibasic cleavage site motif with K/RSSR*GLF being most commonly reported for viruses of the G1 lineage. This is in line with their in vivo characterization as LP [28, 29]. Single H9N2 isolates were detected some time ago in China [30] and Israel [31] which harbored a tribasic motif: PARSRR or PARSKR. Since 2011 until 2018, in Bangladesh, 34 H9N2 isolates have been described which express a tribasic (PAKSKR) HACS motif [32].

In this study, we characterize recent H9 viruses of a wide geographic origin and expressing different HACS motifs with respect to their HA cleavability and pathogenic potential in chicken embryos and juvenile chickens.

## Materials and methods

### Viruses and cells

A total of 22 viruses were obtained from the virus repository of the National Reference Laboratory for Avian Influenza Virus (NRL-AIV) at FLI. Seventeen recent samples originated from a wide geographic area covering Asia, Northern Africa and Europe and are characterized here. The remaining 5 samples have been analyzed earlier. Detailed sample information is available from Additional file 1.

Madin-Darby canine kidney [(MDCK-II) (ATCC^®^ CRL-2936™)], Japanese quail fibroblast [QM9 (CCLV-RIE 466: CVCL_0I49)], chicken embryo fibroblast [DF-1, (ATCC^®^ CRL-12203™)], swine testicle epithelium [ST (ATCC^®^ CRL-1746™)], human pneumocytes [A549 (ATCC^®^ CCL-185™], and human colon epithelial [LoVo (ATCC^®^ CCL-229™)] cell lines were obtained from the bio-bank at FLI. All the cell lines were cultivated in appropriate medium containing 5% of fetal calf serum (FCS).

### Virus isolation and propagation

Before use in this study, viruses were propagated once in 10-days-old specific pathogen free (SPF) embryonated chicken eggs (ECEs) using standard procedures [14]. Hemagglutination assay (HA) was performed using 1% chicken red blood cells (RBC) as described [14].

### Sequencing and phylogenetic analyses

Specific genome segments were amplified using standard RT-PCR [33, 34], amplificates purified and Sanger-sequenced. Sequences were deposited in the Global Initiative on Sharing All Influenza Data (GISAID) database (Additional file 1). Alignment, identity and distance matrices were established using MAFFT [35]. Maximum likelihood phylogenetic analysis was carried out using the IQ Tree software, version 1.6.7 [36, 37]. The HA phylogenetic tree was edited, designed and viewed using the FigTree v1.4.2 software [38] and InkScape 0.92.

### Viral growth characteristics

Plaque assays were performed on MDCK-II cells both in the presence and absence of 2 µg/mL of TPCK-treated trypsin (Sigma T1426) as described [16].

### In silico prediction of endoproteolytic cleavability

Deduced HA protein sequences of low pathogenic (LP) H9 viruses as well as of a highly pathogenic H5N1 virus (considered as a positive control) were analyzed by endoproteolytic cleavage prediction algorithms PiTou [39] and ProP [40].

### In vitro analysis of intracellular proteolytic HACS cleavability

**Figure 1:**
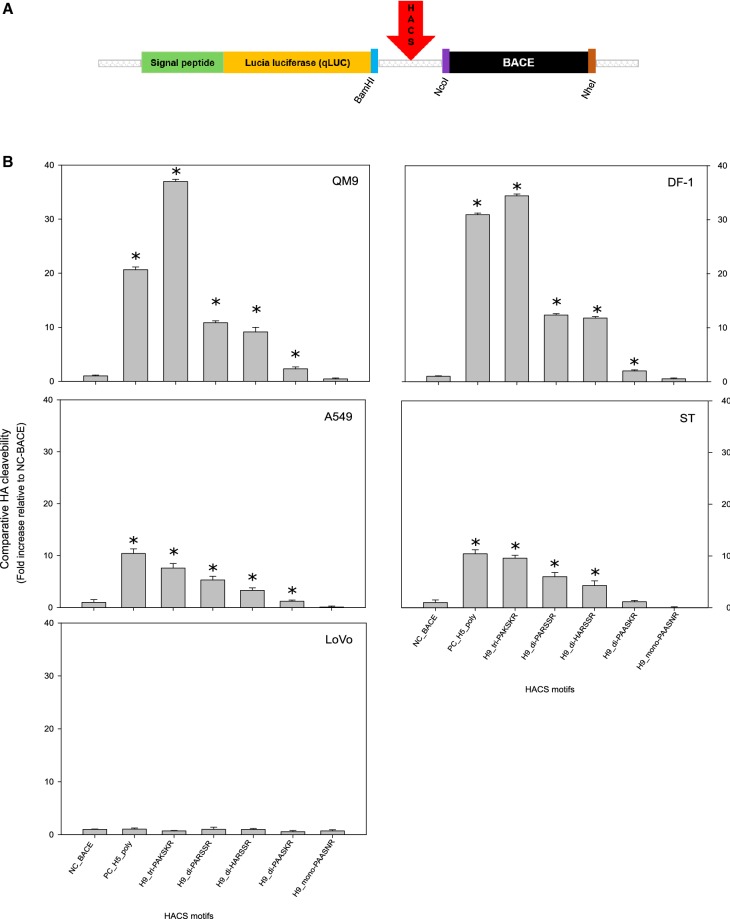
**Assessing the cleavability of the hemagglutinin cleavage site (HACS) of different avian influenza viruses. A** A secretable luciferase protein construct was created by linking LUCIA^®^ luciferase to a trans-Golgi retention anchor (BACE) via an influenzaviral HACS. **B** Luciferase activity in the supernatant of transfected cell cultures is proportional to cleavage at the HACS within the constructs. The constructs were expressed in avian (QM9, DF-1) and mammalian cells (A549, ST). LoVo cells do not express furin activity [51]. Expression levels are compared quantitatively, error bars represent 1 SD from ten independent experiments; *(*p* < 0.05; two-sample *t*-test) indicates a statistically significant difference from the NC-BACE negative control and from other samples. PC_H5_poly represents the polybasic HACS of a highly pathogenic avian influenza virus H5N1 from Bangladesh and is used here as a positive control.

Plasmids were constructed based on the pSELECT-N-Lucia system (InvivoGen, USA) as depicted in Figure 1A. In brief, a secretable luciferase reporter (qLUC) was linked to a trans-Golgi network (TGN) anchor sequence consisting of the C-terminal 71 amino acids of the human beta-secretase 1 isoform B precursor (BACE), residues 406–476 (NCBI accession number NP_620428.1). As a linker of these sequences, an HACS sequence was introduced in frame (Figure 1A). The HACS consisted of amino acids (AA) spanning the motif − P14 to + P5 according to Tian et al. [39]. Sequences of the various H9 HACS as well as negative (BACE only, no HACS) and positive (Bangladeshi HPAIV H5N1) controls are listed in Additional file 2. Different H9 HACS motifs including, tri- di-, and monobasic configurations were cloned for the luciferase assay (Additional file 2). Protein expressed from these constructs under control of the hEF1-HTLV promoter is trafficked to the TGN but would remain anchored there, if no processing occurs at the introduced endoproteolytic cleavage site. However, qLUC is released and secreted into the culture medium when endoproteolytic processing is accomplished. Sequence integrity was confirmed by Sanger sequencing, and plasmids were purified by the QIAfilter™ Plasmid Maxi kit (Qiagen) before transfection.

### qLUC chemoluminescence assay

QM9, DF-1, A549, ST or LoVo cell cultures in 24-well plates were transfected at 70% confluency using Lipofectamin 3000^®^ (Thermo Fisher Scientific) according to the manufacturer’s instructions. At 48 h after transfection, cell-free culture supernatant was collected. Transfection efficiency of the different cell lines was controlled by fluorescence microscopy using a plasmid of comparable size and expressing EGFP.

Chemoluminescence activity in collected supernatants was determined using the QUANTI-Luc™ assay (InvivoGen, USA). In brief, 15 µL of the respective cell-free supernatant were pipetted in triplicate to which 50 µL per well of the QUANTI-Luc™ reagent were added and immediately measured in the Infinite 200 PRO reader (TECAN). Comparison between different cells, transfection efficiencies and cleavage sites were facilitated by use of a negative control (BACE-only, no HACS) for normalization. Fold-increase values were calculated on that base. The two-sample t-test was used to evaluate the average difference between each of two HACS configurations.

### In ovo pathogenesis study

Selected H9 viruses expressing different HACS motifs were inoculated into at least two 14-days old SPF ECEs per sample and incubated for 96 h. Each embryo was carefully removed off the egg by cutting one egg pole with scissors and was subsequently submerged in 4% neutral buffered formaldehyde (NBF). The remaining egg shell was cut in two sections and the CAM was separated from the inner egg shell surface and spanned onto an eyelet (Ø = 14 mm, PÖSAMO VAUKA, Monheim am Rhein, Germany) as described elsewhere [41] before fixation in NBF. The prepared CAM-eyelets and each half of a longitudinally sectioned embryo were embedded in paraffin wax. The paraffinized CAM-membranes were then stamped out by using a hole puncher (Ø = 10 mm, S&R Industriewerkzeuge GmbH, Gundelfingen, Germany) to create a round paraffin block of 5 mm thickness. This block was divided into two halves and each part was rotated about 90° followed by a second round of paraffin wax embedding. Finally, 2–4 µm sections were generated and stained with hematoxylin and eosin to investigate the following organs: CAM, nasal cavity, oral cavity, lungs, air sacs, heart, proventriculus, gizzard, intestine, pancreas, liver, kidneys, gonads, spleen, skin, brain. Specimens were evaluated for histopathologic lesions using an Axio Imager M2 microscope (Carl Zeiss Microscopy).

To visualize AIV-matrixprotein, immunohistochemistry (IHC) was performed using the avidin–biotin–peroxidase-complex (ABC) method essentially as described [27]. The distribution of AIV-matrixprotein was semiquantitatively assessed for each organ by scoring on a 0 to 3 scale: 0 = negative; 1 = focal or oligofocal, 2 = multifocal, 3 = coalescing to diffuse immunoreactive cells as previously described [27].

### In vivo study (Intravenous Pathogenicity Index, IVPI)

White Leghorn chickens hatched at FLI from SPF ECE and purchased from Lohmann Animal Health, Cuxhaven, Germany, were raised until they were 6 weeks old. A total of 10 chickens were used for each virus isolate. Five H9Nx isolates representative of different HACS configurations and or genotypes were selected for the IVPI. The IVPI was essentially conducted as described in OIE manual [14].

## Results

### Sequence analyses and phylogeny

**Figure 2:**
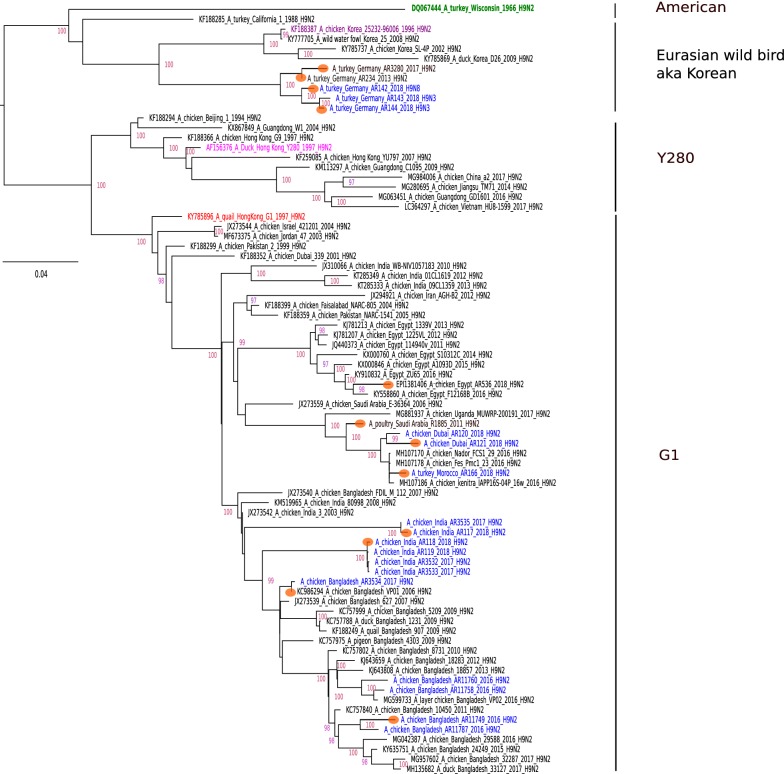
**Maximum likelihood phylogeny of deduced H9 hemagglutinin protein sequences.** The maximum likelihood tree was established using the IQ-Tree software. Robustness of branching orders was tested by an ultrafast bootstrap approximation algorithm [36]. The tree is drawn to scale and color-coded clading information is shown to the right of the tree. Blue color indicates sequences that were established in the frame of this study. Orange circles depict H9 viruses for further experiments in this study.

Analyses of the HA and NA sequences of 17 recent H9 AIV isolates of poultry from various Asian, African and European countries revealed the presence of viruses of subtypes H9N2, H9N3 and H9N8 (Additional file 1). By maximum likelihood phylogeny, the HAs were shown to cluster with geographical and temporal restrictions (Figure 2). Except for viruses of German origin which clustered within the Eurasian wild bird (aka Korean) lineage, all viruses belonged to the G1 lineage. G1 viruses segregated into further branches with considerable genetic distance between the branches. The HA proteins harbored different HACS motifs (representatives of each type are shown in Table 1). In phylogenetically closely related G1 viruses from Bangladesh and India, the tribasic motif PAKSKR was detected. In addition, several dibasic motifs, PARSSR, HARSSR, and PAKSSR were identified for other examined G1 viruses. In H9 viruses from Germany, monobasic HACS were encoded: PAASSR, PAASNR. The NA sequences confirmed that the origin of subtype N2 was embedded in the G1 lineage. The German N3 and N8 segments were closely related to European wild bird subtypes H2N3, H7N3, H10N3 and H3N8, respectively.

**Table 1:** **Endoproteolytic cleavage site of the hemagglutinin protein (HACS), in silico prediction and in vitro measurement of furin-mediated cleavage and trypsin dependency of cell culture growth of several H9Nx avian influenza viruses**

Viruses^a^	Subtypes	Deduced HACS sequences		ProP score^b^	PiTou score^c^	Quanti-Luc assay^d^		Trypsin-independent growth
		Motif-P14 to -P1	Motif type			Avian cells^e^	Mammalian cells^f^	
A/duck/Bangladesh/AR132-D1/2016 (BD_132-D1)	H5N1 (positive control)	LATGLRNSPQRE*RRRKR*	Polybasic	***0.771***	***369***	26 ± 0.3	10 ± 0.7	Yes
BD_11749	H9N2	LAIGLRNVPA*K*S*KR*	Tribasic	***0.628***	***376***	35 ± 0.3	9 ± 0.6	No
BD_11758				***0.628***	***376***	N/D^g^	N/D	N/D
BD_11760				***0.628***	***376***	N/D	N/D	N/D
BD_11787				***0.628***	***376***	N/D	N/D	N/D
IN_117				***0.628***	***376***	N/D	N/D	No
IN_3535				***0.628***	***376***	N/D	N/D	N/D
IN_118	H9N2	LAIGLRNVPA*R*SS*R*	Dibasic-1	0.344	***366***	11 ± 0.2	6 ± 0.7	No
IN_119				0.344	***366***	N/D	N/D	N/D
IN_3532				0.344	***366***	N/D	N/D	N/D
IN_3533				0.344	***366***	N/D	N/D	N/D
EG_536				0.344	***366***	N/D	N/D	N/D
BD_VP01	H9N2	LAIGLRNVPA*K*SS*R*	Dibasic-2	0.063	− 175	N/D	N/D	No
BD_3534				0.063	− 175	N/D	N/D	N/D
DU_120	H9N2	LAIGLRNVQA*R*SS*R*	Dibasic-3	0.325	***213***	N/D	N/D	No
MO_166	H9N2	LAIGLRNV*H*A*R*SS*R*	Dibasic-4	0.324	***320***	10 ± 0.5	4 ± 0.6	No
DU_121				0.324	***320***	N/D	N/D	No
SA_1885				0.324	***320***	N/D	N/D	N/D
DE_3280	H9N2	LAVGLRNVPAAS*KR*	Dibasic-5	0.375	178	2 ± 0.2	1 ± 0.2	N/D
DE_234	H9N2	LAVGLRNVPAASN*R*	Monobasic-1	0.054	− 490	0.4 ± 0.1	0.1 ± 0.1	No
DE_143	H9N3			0.054	− 490	N/D	N/D	No
DE_144	H9N3			0.054	− 490	N/D	N/D	No
DE_142	H9N8	LAVGLRNVPAASS*R*	Monobasic-2	0.054	− 490	N/D	N/D	No
pSelect-BACE^h^	Negative control	DKIKGLAGDRGGGSSAAAMV	N/D	< 0.08	− 612	1 ± 0.05	1 ± 0.4	N/D

Viruses are listed according to the HACS sequence type.

Italicized font indicates the basic amino acids.

^a^Abbreviations indicate country of origin (two letters) and number of isolate. Full names, origin etc. are presented in Additional file 1.

^b^Thresholds of software-assisted cleavage prediction of > 0.5 (ProP) and.

^c^> 185 (PiTou), respectively, indicate likelihood of intracellular cleavage by furin-like endoproteases (bolditalics).

^b,c^Similar values, within a motif type simply indicate that the same HACS sequence of different viruses has been tested.

^d^Fold increase of luciferase release from cells expressing a protein construct that links secretable luciferase via the respective cleavage site to a trans-Golgi anchor motif (see Figure 1A).

^e^Avian cells—QM-9, DF-1.

^f^Mammalian cells—A549, ST.

^g^N/D—not done/tested/applicable.

^h^pSelect-BACE construct lacking any proteolytic cleavage site (used as a negative control in luciferase assay).

Sequence analyses of internal genome segments obtained for viruses of the G1 lineage and Korean lineage of German isolates revealed homogenous genotype patterns related to the HA lineage (Additional file 3). The exception was a single isolate from Bangladesh (BD_11749), which harbored a PB2 gene originating from HPAIV H5 clade 2.3.2.1a with considerable genetic distance to the PB2 of other studied H9N2 viruses. Mutations or motifs of internal genome segments reported to be associated with either increased polymerase activity or pathogenicity or promoting mammalian adaptation are summarized in Additional file 4.

### HACS processing characteristics

As the composition of the HACS, not only of subtype H5 and H7 AIVs, may determine pathogenicity in chickens, special attention was addressed to the mono-, di- and tribasic configurations of the H9 HACS of the viruses under this study. Firstly, PiTou and ProP furin cleavage prediction algorithms [39, 40] were employed for in silico analyses. Results shown in Table 1 revealed that the predicted likelihood of furin-assisted cleavability increased with the number of basic AA. The H9 tribasic and the H5 polybasic motifs concordantly were predicted highly cleavable. Some H9 dibasic motifs scored above threshold in the PiTou method only.

These results were counterchecked in an in vitro cleavage assay (qLUC assay) in which the concentration of secreted luciferase was proportional to the intracellular endoproteolytic processability of the HACS (Table 1, Figure 1B). In this sequence context, the H9 tribasic HACS PAKSKR was processed at least as efficiently as the H5 polybasic HACS (Table 1, Figure 1B). Two dibasic motifs, PARSSR and HARSSR were processed also, yet at significantly (*p* < 0.05) lower efficiency as the above; these cleavage sites were predicted processible by the PiTou algorithm only. Another dibasic motif (PAASKR) and a monobasic HACS were not processed. Processing was significantly (*p* < 0.05) more efficient in avian (QM-9, DF-1) compared to mammalian cell lines (A549, ST); the furin-deficient LoVo cell line failed to process any of the constructs (Figure 1B). Thus, in silico and in vitro an influence on furin-assisted processability of the number of basic AA at the isolated H9 HACS was verified (Table 1).

### Trypsin dependency of viral growth in cell culture

HPAIVs with a polybasic HACS are capable of trypsin-independent replication in cell culture. Plaque assays on MDCK-II cells showed that all studied H9 isolates required trypsin for the formation of visible plaques (Table 1). This included BD_11749 and IN_117 expressing a tribasic HACS (PAKSKR) and found to be readily processed in the qLUC assay. In contrast, the HPAIV H5N1 (BD_132-D1) encoding a polybasic HACS (RERRRKR) formed plaques in the absence of exogenous trypsin.

### In ovo pathogenicity

**Figure 3:**
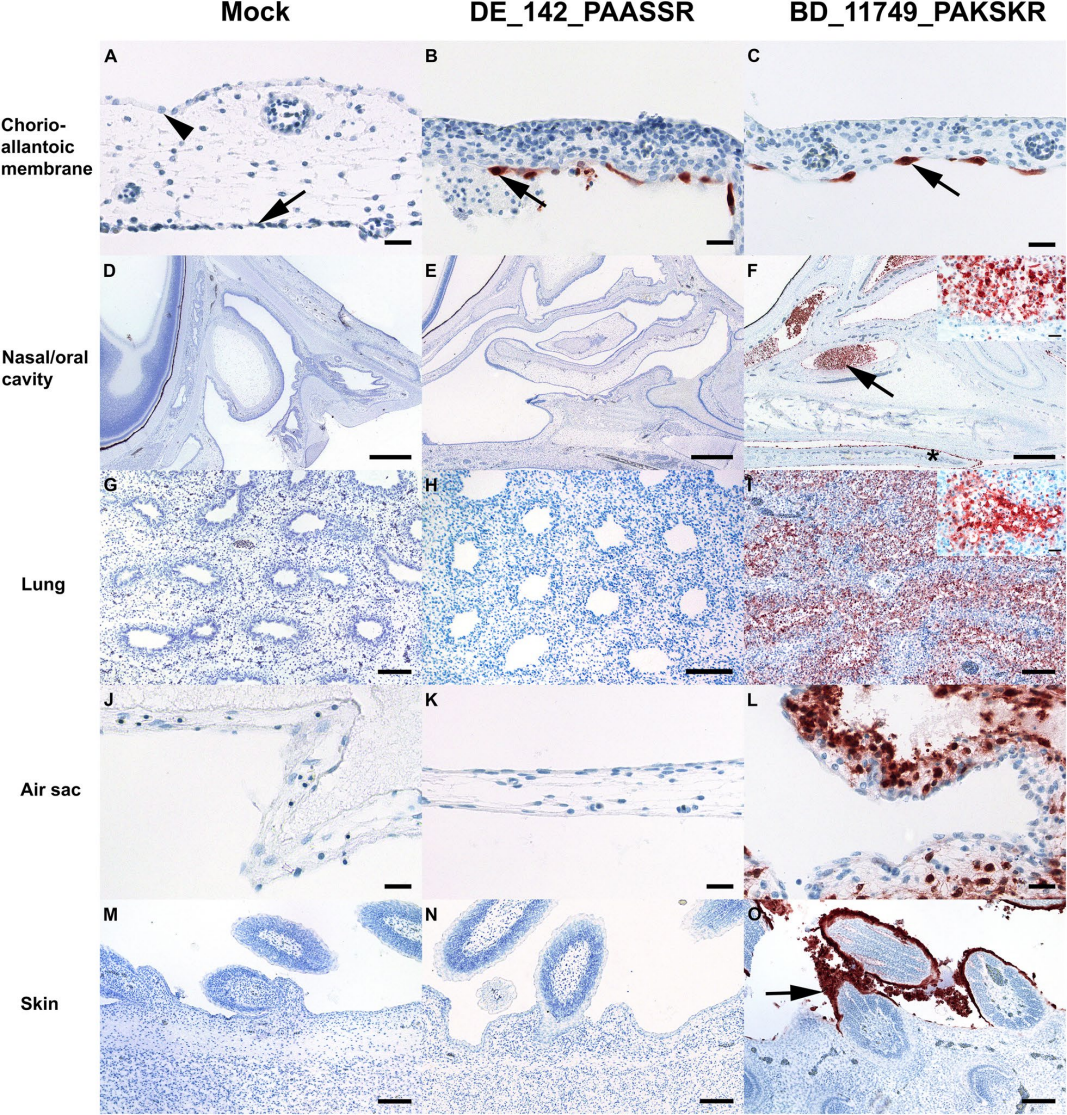
**Immunohistological findings in mock (*n* = 2), DE_142-PAASSR (*n* = 2) and BD_11749_PAKSKR (*n* = 3) avian influenza virus (AIV) infected 14** **days old chorio-allantoic membranes and chicken embryos. A** Normal cuboidal epithelium (arrowhead) of the chorion and flattened epithelium (arrow) of the allantois. **B**, **C** Intense matrixprotein immunoreactivity confined to allantoic epithelium (arrow). **D**, **E** Nasal cavity and a section of the eye in the left upper corner. **F** Necrotizing rhinitis with marked luminal accumulation of matrixprotein positive debris in the rostral (arrowhead) and caudal nasal chamber as well as immunoreactive squamous epithelium of the tongue (star); Inset: The lumen of the nasal cavity is filled by epithelial debris and remaining respiratory epithelium is either flattened or shows signs of nuclear pyknosis and karyorrhexis. **G**, **H** Lung with non-altered parabronchi. **I** Necrotizing (para-) bronchiolointerstitial pneumonia; Inset: intense matrixprotein positive parabronchiolar epithelium and debris. **J**, **K** Air sac membrane lined by simple squamous epithelium. **L** Necrotizing airsacculitis with many degenerating and strong matrixprotein positive epithelial cells. (M, N). Epidermal squamous epithelium and few follicle buds. (O) Intensely matrixprotein positive squamous and partially degenerating epithelium (arrow). AIV-matrixprotein immunohistochemistry, avidin–biotin–peroxidase complex method with 3-amino-9-ethyl-carbazol as chromogen and hematoxylin counterstain. Bars; **A**–**C**, **J**–**L**, Inset **F**, **I** = 20 µm; **D**–**F** = 500 µm; **G**–**I**, **M**–**O** = 100 µm.

Based on previous data [27, 42, 43] embryonated chicken eggs (ECE) were chosen as an easily accessible and informative in vivo system to assess AIV pathogenicity. Expression of matrixprotein (M) in infected ECE was semiquantitatively assessed in the chorioallantoic membrane (CAM) and in various embryonic organs by immunohistochemistry (Additional file 5). No histopathologic lesions or M antigen were found in mock-infected embryonic organs or CAM (Figures 3A, D, G, J, M). All H9 viruses studied replicated in the CAM as evidenced by oligo- to multifocal formation of M protein positive cells exclusively confined to the allantoic epithelium (Figures 3B, C). Interestingly, the BD_AR11749 and IN_117 H9N2 virus that expressed a tribasic HACS motif showed extended replication to various embryonic organs. Alterations were seen in nasal and oral cavity and were characterized by a moderate to severe, acute, multifocal, necrotizing rhinitis, glossitis, or stomatitis with epithelial sloughing and accumulation of intra luminal debris (Figure 3F). Mild, multifocal to coalescing and necrotizing alterations were seen mostly in few layers of squamous epithelium of the epidermis and feather buds (Figure 3O). In one of the IN_117 infected embryos necrotizing lesions extended into the lungs and air sacs with diffuse and intense M protein positive epithelial cells and debris (Figure 3I, L). Furthermore, extensive M protein positive debris was seen in the proventriculus and gizzard (Additional file 5) and were interpreted as ingested debris derived from the upper and lower respiratory tract. In one out of three embryos inoculated with BD_11749 oligofocal M antigen expression in squamous epithelium of the skin was noticed.

A slightly wider tissue invasion was also evident for isolate EG_536 and IN_118 encoding a dibasic HACS (Additional file 5). In three out of three chicken embryos inoculated with the dibasic HACS isolate BD_VP01 oligo- to multifocal squamous epithelial skin cells, mostly confined to the apical layer of epidermis, showed intense M antigen expression which extended in one out of three into the oral and nasal cavity (Additional file 5). Another two dibasic HACS isolates, MO_166 and DU_121, which showed endoproteolytical processing in the qLUC assay, however, were indistinguishable from the monobasic H9 virus DE_142 (Figures 3B, E, H, K, N; Additional file 5) for which viral replication was confined to the allantoic epithelium of the CAM.

Endothelial localization of M antigen associated with widespread parenchymal necrosis as typically seen in replication of H5 and H7 HP phenotypes in ECE was not observed for any of the H9 isolates.

### Pathogenicity in juvenile chickens

In vivo pathogenicity was also assessed in 6 weeks old chickens inoculated via the intravenous route and examined for clinical signs over a period of 10 days. All inoculated chickens survived without showing any clinical signs, thus the IVPI score remained zero for all studied viruses (Table 2). Seroconversion rates at day 10 post-inoculation were homogenous among the groups with the exception of the group inoculated with isolate DE_142 (H9N8) which showed a noticeably lower seroconversion rate and lower HI titers (Additional file 6). Consequently and independently of the distinct HACS and genome constellations all tested H9 viruses have to be considered as low pathogenic.

**Table 2:** **Analyses of virulence in ovo, in vivo and substituting mutations in HA and NA glycoproteins described to increase virulence of H9Nx strains in the chicken host [**19**]**

Strains/isolates	Subtypes	Deduced HACS sequence		Virulence^a^ in ovo	In vivo (IVPI)	HA			NA
		Motif-P6 to -P1	Motif type			E198A	Q234L	E502D	V33M
BD_11749	H9N2	PA*K*S*KR*	Tribasic	20	0	A	L	E	M
BD_11758				N/D	N/D	A	L	Q	M
BD_11760				N/D	N/D	A	L	E	M
BD_11787				N/D	N/D	A	Q	E	M
IN_117				20	N/D	A	L	E	M
IN_3535				N/D	N/D	A	L	Q	M
IN_118	H9N2	PA*R*SS*R*	Dibasic-1	17	0	A	L	E	M
IN_119				N/D	N/D	A	L	E	M
IN_3532				N/D	N/D	A	L	E	M
IN_3533				N/D	N/D	A	L	E	M
EG_536				17	N/D	A	L	E	M
BD_VP01	H9N2	PA*K*SS*R*	Dibasic-2	8	0	A	L	E	M
BD_3534				N/D	N/D	A	L	E	M
DU_120	H9N2	QA*R*SS*R*	Dibasic-3	N/D	N/D	A	Q	E	M
DU_121	H9N2	*H*A*R*SS*R*	Dibasic-4	2	N/D	T	L	E	M
MO_166				2	0	T	R	E	M
SA_1885				N/D	N/D	T	L	E	M
DE_3280	H9N2	PAAS*KR*	Dibasic-5	N/D	N/D	E	Q	E	M
DE_234	H9N2	PAASN*R*	Monobasic-1	2	N/D	E	Q	E	M
DE_143	H9N3			N/D	N/D	E	Q	E	N/A
DE_144	H9N3			N/D	N/D	E	Q	E	N/A
DE_142	H9N8	PAASS*R*	Monobasic-2	2	0	E	Q	E	N/A
Mock^b^	N/A	N/A	N/A	0	N/A	N/A	N/A	N/A	N/A

Italicized font indicates the basic amino acids.

N/D: not determined, N/A: not applicable.

^a^Virulence was tested by semi-quantifying viral spread in embryonic tissues. The values represent a cumulative score based on immunohistochemical investigation of 17 different tissues; higher values indicate extended tissue spread. See Additional file 5 for scoring details.

^b^SPF allantoic fluid.

## Discussion

H9 AIV isolated from recent poultry samples from Asia, Africa, and European countries revealed subtypes H9N2, H9N3, and H9N8, most of them clustering with the G1 lineage, with HA proteins harboring mono-, di-, or tri-basic cleavage site motifs. Tribasic cleavage site motifs are unusual in low pathogenic AIV and, so far, have only been detected in viruses of the H9 subtype. In subtypes H5 and H7 the number of basic amino acids at the HACS is an important determinant of pathogenicity. Our study has added to the knowledge on molecular and pathogenic properties of these recently isolated H9 viruses as follows:

1. The tribasic H9 HACS as well as two dibasic motifs (PARSSR, HARSSR) are processable at significantly higher levels than any monobasic H9 HACS by furin-like endoproteases as predicted in silico and shown in vitro using a newly developed qLUC assay (Figure 1B). Processing of the HACSs in the ectopic sequence context of the qLUC assay fully mirrored in silico HACS cleavage prediction by the PiTou [39] but not by the ProP [40] method. The two prediction tools operate on a different base invoking either complex hidden Markov models [38] or neural networks [39], which may explain the discrepant results. The ProP method was described to have a lower sensitivity for cleavage by some proprotein convertases [40]. When tested in the qLUC assay avian cells processed the HACS more efficiently than mammalian ones. Enhanced furin-dependent cleavage activation by chicken-derived furin of an H9 G1 virus with a tribasic HACS was previously demonstrated [44] with processing being dependent on intracellular furin expression levels.
2. Furin processability of tribasic ectopic H9 HACSs did not translate into trypsin-independent growth in MDCK cells. A truly polybasic HACS, such as that of H5/H7 highly pathogenic isolates, is cleaved intracellularly by furin-like proteases leading to the incorporation of mature, cleaved HA1, 2 into budding virions which allows for multiple rounds of infections in cell culture independent of external trypsin supplementation. Obviously the H9 HACSs when in their homotopic sequence context in the HA protein were inaccessible to proprotein convertases and remained uncleaved. Sterical hinderance of furin accessibility to the HACS has previously been implicated for several H9 viruses [44]. Grafting an additional basic amino acid into the HACS of a H9N2 strain to produce the sequence RSRR, similar to the tribasic ones examined in our study, did not invoke trypsin-independent growth nor did it increase in vivo pathogenicity; only the insertion of the complete polybasic HACS of an HP H5 virus (RRRKKRR) achieved conversion to the HP phenotype [26]. Similarly, another H9N2 virus expressing the engineered HACS sequence RKKR [45] was not capable of trypsin-independent growth, and serial passaging in air sacs of chickens was required to select a variant, which had acquired a number of coding mutations in various virus genes to promote trypsin-independent growth and increased pathogenicity in vivo [45]. Recent research results on influenza virus activating host proteases point towards intricate subtype- and host-specific features [46, 47]. Therefore, extrapolation of results obtained by cleavage prediction algorithms and the qLUC assay to the cellular level of viral replication remain difficult.
3. Extended tissue invasion in infected chicken embryos, indicative of enhanced pathogenicity, was observed with H9 viruses harboring a tribasic HACS (BD_11749 and IN_117, Table 2, Figure 3, Additional file 5). Endothelial and CNS infection, a hallmark of true HPAIV infection in the in ovo model [27], however, was not evident for any of the H9 isolates. Tissue tropism varied greatly with H9 viruses expressing different dibasic HACS motifs (EG_536, IN_118, BD_VP01, DU_121 and MO_166) and reflected a highly variable pathogenic potential of these viruses (Table 2; Additional file 5). Isolates with a monobasic HACS (DE_142, DE_234) remained strictly confined to the allantoic epithelium (Figure 3, Additional file 5), as seen in typical LPAIV infections [43]. Likewise, in the standard in vivo model of pathogenicity evaluation in juvenile chickens (providing matured immune defense functions), none of the examined H9 viruses caused any clinical signs. This justifies classification of all isolates examined to be of a low pathogenic phenotype. Pathogenicity of AIV is a polygenic trait. Although, in subtypes H5 and H7, the configuration of the HACS has a major influence on pathogenicity, other modifying factors can reside within the HA as well as in other viral proteins [45, 48, 49]. As a limitation of this study, isogenic viruses differing only in the HACS sequences were not available in this study; thus, a strict correlation between the enhanced furin-associated HACS processability detected in the qLUC assay and pathogenicity in vivo could not be examined in detail. The viruses studied actually differed not only in their HACS but also with respect to their genome constellation (Additional file 3) affecting evens the subtype (N2, N3, N8). Therefore, further traits residing in the HA and other viral proteins may have played a role in the intermediate pathogenicity observed for isolates with a tribasic HACS in the in ovo model. Sequence analyses of the internal gene segments revealed a number of substituting mutations described to be related to pathogenicity (Additional file 4). However, no correlation of these patterns with the in ovo results was evident. Recently another mechanism of increasing pathogenicity has been reported specifically in H9 viruses [19]: presence of four substitutions in the HA (E198A, Q234L, E502D; H9 numbering in the present study) and NA (V33M) distinguished a plaque-cloned G1 H9N2 with extended pathogenicity in 4 weeks old SPF chickens [19]. Several of our examined isolates showed three of those four substitutions (Table 2) including BD_11749 and IN_117 which also harbored a tribasic HACS and expressed the highest pathogenicity score in the in ovo system (Table 2). However, deciphering the influence of those mutations and their interplay with different HACS configurations will require the generation of recombinant viruses by reverse genetics in future studies.

AIV H9 infections of chickens in Asia, the Middle East and Africa are often associated with secondary infections resulting in high morbidity and mortality [50]. This blurs a clinical demarcation from infections with true HPAIV, and the detection at increasing frequency in southern Asia of H9N2 viruses encoding additional basic AA in their HACS is alarming. Until to date the only non-H5/H7 AI viruses that naturally express a tribasic HACS are found in the H9N2 G1 lineage and are geographically restricted, so far, to southern Asia. Considering the widespread endemic status for the zoonotic H9N2 G1 lineage in poultry populations further spread also of variants with potentially increased (intermediate) pathogenicity must be expected. From a One Health aspect it would be particularly important to limit the spread of such AI viruses.

## Supplementary information

**Additional file 1. List of viruses, including sequence accession numbers, used in this study.**

**Additional file 2. Primers designed for cloning and sequencing of the HACS of H9Nx viruses.**

**Additional file 3. Genotype determination of H9Nx viruses.**

**Additional file 4. Analyses of substituting mutations described to increase pathogenicity and promoting mammalian adaptation of H9Nx viruses fully sequenced in this study.**

**Additional file 5. Distribution in tissues of matrix protein immunoreactivity in mock and H9Nx infected chicken embryos assessed by semiquantitative scoring (0 = negative; 1 = focal/oligofocal; 2 = multifocal; 3 = coalescing/diffuse) and expressed as median values (M) with upper limit (UL) respectively lower limit (LL).**

**Additional file 6. Pathogenicity indices and seroconversion of ten chickens each after intravenous inoculation of H9Nx viruses.**

## Data Availability

Sample information and the accession number of the sequences that deposited in the Global Initiative on Sharing All Influenza Data (GISAID) database are available in (Additional file 1).
