# Supplementary material for: Comparison of pathogenicity of subtype H9 avian influenza wild-type viruses from a wide geographic origin expressing mono-, di-, or tri-basic hemagglutinin cleavage sites
Source: Vet Res. 2020 Mar 31;51:48. doi: 10.1186/s13567-020-00771-3 (PMC7106749; doi:10.1186/s13567-020-00771-3)
Supplement: Supplementary file 1 — Additional file 1. List of viruses, including sequence accession numbers, used in this study. [file 13567_2020_771_MOESM1_ESM.docx]

**Additional file 1**. List of viruses, including sequence accession numbers, used in this study.

|  | **Strains/isolates** | **Short form** | **Deduced HACS sequence** | | **Accession number (EpiFlu database; GenBank)** | | | | | | | |
| --- | --- | --- | --- | --- | --- | --- | --- | --- | --- | --- | --- | --- |
|  |  |  | **Motif -P6 to -P1** | **Motif type** | PB2 | PB1 | PA | HA | NP | NA | M | NS |
| 1 | A/chicken/Bangladesh/AR11749/16 (H9N2) | BD_11749 | PAKSKR | Tribasic | EPI1508517 | EPI1508518 | EPI1508519 | EPI1508520 | EPI1508521 | EPI1508522 | EPI1508523 | EPI1508524 |
| 2 | A/chicken/Bangladesh/AR11758/16 (H9N2) | BD_11758 |  |  | EPI1508525 | EPI1508526 | EPI1508527 | EPI1508528 | EPI1508529 | EPI1508530 | EPI1508531 | EPI1508532 |
| 3 | A/chicken/Bangladesh/AR11760/16 (H9N2) | BD_11760 |  |  | EPI1508533 | EPI1508534 | EPI1508535 | EPI1508536 | EPI1508537 | EPI1508538 | EPI1508539 | EPI1508540 |
| 4 | A/chicken/Bangladesh/AR11787/16 (H9N2) | BD_11787 |  |  | EPI1508541 | EPI1508542 | EPI1508543 | EPI1508544 | EPI1508545 | EPI1508546 | EPI1508547 | EPI1508548 |
| 5 | A/chicken/India/AR117/18 (H9N2) | IN_117 |  |  | EPI1555330 | EPI1555457 | EPI1555557 | EPI1555583 | EPI1555700 | EPI1555734 | EPI1555809 | EPI1555816 |
| 6 | A/chicken/India/AR3535/17 (H9N2) | IN_3535 |  |  | EPI1555107 | EPI1555218 | EPI1555221 | EPI1555236 | EPI1555308 | EPI1555317 | EPI1555318 | EPI1555319 |
| 7 | A/chicken/India/AR118/18 (H9N2) | IN_118 | PARSSR | Dibasic-1 | EPI1555978 | EPI1556168 | EPI1556205 | EPI1556230 | EPI1556271 | EPI1556272 | EPI1556279 | EPI1556280 |
| 8 | A/chicken/India/AR119/18 (H9N2) | IN_119 |  |  | EPI1556427 | EPI1556587 | EPI1556614 | EPI1556708 | EPI1556744 | EPI1556773 | EPI1556795 | EPI1556811 |
| 9 | A/chicken/India/AR3532/17 (H9N2) | IN_3532 |  |  | EPI1553400 | EPI1553413 | EPI1553422 | EPI1553431 | EPI1553441 | EPI1553450 | EPI1553455 | EPI1553461 |
| 10 | A/chicken/India/AR3533/17 (H9N2) | IN_3533 |  |  | EPI1553909 | EPI1553923 | EPI1553935 | EPI1553947 | EPI1553960 | EPI1553978 | EPI1554167 | EPI1554177 |
| 11 | A/chicken/Egypt/AR536/18 (H9N2)* | EG_536 |  |  | N/D | N/D | N/D | EPI1381406 | N/D | N/D | N/D | N/D |
| 12 | A/chicken/Bangladesh/AR3534/17 (H9N2) | BD_3534 | PAKSSR | Dibasic-2 | EPI1554348 | EPI1554513 | EPI1554563 | EPI1554590 | EPI1554615 | EPI1554729 | EPI1554932 | EPI1554960 |
| 13 | A/chicken/Bangladesh/VP01/06 (H9N2)* | BD_VP01 |  |  | KC986287 | KC986288 | KC986289 | KC986294 | KC986291 | KC986292 | KC986293 | KC986290 |
| 14 | A/chicken/Dubai/AR120/18 (H9N2) | DU_120 | QARSSR | Dibasic-3 | EPI1557005 | EPI1557035 | EPI1557088 | EPI1557113 | EPI1557130 | EPI1557133 | EPI1557135 | EPI1557148 |
| 15 | A/chicken/Dubai/AR121/18 (H9N2) | DU_121 | HARSSR | Dibasic-4 | EPI1564146 | EPI1564147 | EPI1564148 | EPI1564149 | EPI1564150 | EPI1564151 | EPI1564152 | EPI1564153 |
| 16 | A/turkey/Morocco/AR166/18 (H9N2) | MO_166 |  |  | EPI1564154 | EPI1564155 | EPI1564156 | EPI1564157 | EPI1564158 | EPI1564159 | EPI1564160 | EPI1564161 |
| 17 | A/poultry/Saudi Arabia/R1885/11 (H9N2)* | SA_1885 |  |  | N/D | N/D | N/D | EPI1564304 | N/D | N/D | N/D | N/D |
| 18 | A/turkey/Germany/ AR3280/17 (H9N2)* | DE_3280 | PAASKR | Dibasic-5 | N/D | N/D | N/D | EPI1564305 | N/D | N/D | N/D | N/D |
| 19 | A/turkey/Germany/AR234/13 (H9N2)* | DE_234 | PAASNR | Monobasic-1 | N/D | N/D | N/D | EPI241962 | N/D | N/D | N/D | N/D |
| 20 | A/turkey/Germany/AR143/18 (H9N3) | DE_143 |  |  | EPI1564170 | EPI1564171 | EPI1564172 | EPI1564291 | EPI1564292 | EPI1564293 | EPI1564294 | EPI1564295 |
| 21 | A/turkey/Germany/AR144/18 (H9N3) | DE_144 |  |  | EPI1564296 | EPI1564297 | EPI1564298 | EPI1564299 | EPI1564300 | EPI1564301 | EPI1564302 | EPI1564303 |
| 22 | A/turkey/Germany/AR142/2018 (H9N8) | DE_142 | PAASSR | Monobasic-2 | EPI1564162 | EPI1564163 | EPI1564164 | EPI1564165 | EPI1564166 | EPI1564167 | EPI1564168 | EPI1564169 |

N/D: not done/not available

*Virus isolates previously characterized in the frame of other studies [34, 54].
