# Supplementary material for: Comparison of pathogenicity of subtype H9 avian influenza wild-type viruses from a wide geographic origin expressing mono-, di-, or tri-basic hemagglutinin cleavage sites
Source: Vet Res. 2020 Mar 31;51:48. doi: 10.1186/s13567-020-00771-3 (PMC7106749; doi:10.1186/s13567-020-00771-3)
Supplement: Supplementary file 2 — Additional file 2. Primers designed for cloning and sequencing of the HACS of H9Nx viruses. [file 13567_2020_771_MOESM2_ESM.docx]

**Additional file 2.** Primers designed for cloning and sequencing of the HACS of H9Nx viruses.

| **Target** | **Source** | **Primer** | **Sequences** | **Size^1^ (bp)** |
| --- | --- | --- | --- | --- |
| BACE 1 | Human RNA_A549  cell | Fw-BACE V430_NcoI | CTAC CCA TGG TGGAAGGCCTTTTGTCAC | 213 |
|  |  | Rv-BACE_NheI | CTAA GCT AGC CCTTCAGCAGGGAGATGTCAT |  |
| BACE 2 | DNA_Grap plasmid | Fw-BACE Grap_NcoI | CTAC CCA TGG TTGGACATGGAAGACTGTGG | 198 |
|  |  | Rv-BACE_NheI | CTAA GCT AGC CCTTCAGCAGGGAGATGTCAT |  |
| H9_HACS | DE_3280 | AR3280_  BamHI_Fw | CTAA GGA TCC CTGGCAATCGGCCTGAGA | 57 |
|  |  | AR3280_  NcoI_Rv | CTAA CCA TGG CACCAAACAATCCTCTC |  |
|  | DE_234 | AR234_  BamHI_Fw | CTAA GGA TCC TTGGCAATCGGTCTAAGAAATG | 57 |
|  |  | AR234_  NcoI_Rv | CTAA CCA TGG CACCAAACAATCCTCTATT |  |
|  | SA_1885 | R1885_  BamHI_Fw | CTAA GGA TCC CTGGCAGTCGGCTTGAG | 57 |
|  |  | R1885_  NcoI_Rv | CTAA CCA TGG CTCCGAATAGTCCTCTAC |  |
|  | BD_11749 | AR11749_  BamHI_Fw | CTAA GGA TCC CTAGCAATAGGTCTGAGAAAC | 57 |
|  |  | AR117_  BamHI_Fw | CTAA GGA TCC CTGGCAGTCGGCTTGAG |  |
|  | IN_117 | AR117_  NcoI_Rv | CTAA CCA TGG CCCCAAATAGTCCTCTC | 57 |
|  |  | AR11749_  NcoI_Rv | CTAA CCA TGG CTCCAAATAATCCTCTCTT |  |
|  | IN_118 | AR234_  BamHI_Fw | CTAA GGA TCC TTGGCAATCGGTCTAAGAAATG | 57 |
|  |  | AR118_  NcoI_Rv | CTAA CCA TGG CTCCAAATAGACCTCTAC |  |
|  | MO_166 | AR166_  BamHI_Fw | CTAA GGA TCC CTGGCAATTGGCCTTAG | 57 |
|  |  | R1885_  NcoI_Rv | CTAA CCA TGG CTCCGAATAGTCCTCTAC |  |
| H5_HACS | BD_132-D1 | AR132_  Fw_BamHI | CTCT GGA TCC AACAAATTAGTCCTTGCGACT | 77 |
|  |  | AR132_  Rv_NcoI | CTAA CCA TGG CTCCAAACAATCCTCTTTTTC |  |
| Sequencing primer | pSelect-NLucia-zeo | pSelect_Fw | CTT CCC CAG AGG TGT ACC A | 292 |
|  |  | pSelect_Rv | GTT GTT AAC TTG TTT ATT GCA GCT |  |

^1^ Size of the amplificate.

Colors indicate the corresponding tagged restriction sites: Cyan – *Nco*I, yellow – *Nhe*I, green – *BamH*I
