# Supplementary material for: Comparison of pathogenicity of subtype H9 avian influenza wild-type viruses from a wide geographic origin expressing mono-, di-, or tri-basic hemagglutinin cleavage sites
Source: Vet Res. 2020 Mar 31;51:48. doi: 10.1186/s13567-020-00771-3 (PMC7106749; doi:10.1186/s13567-020-00771-3)
Supplement: Supplementary file 3 — Additional file 3. Genotype determination of H9Nx viruses. [file 13567_2020_771_MOESM3_ESM.docx]

**Additional file 3.** Genotype determination of H9Nx viruses.

| **Strains/Isolates** | **Genotype** | | | | | | | |
| --- | --- | --- | --- | --- | --- | --- | --- | --- |
|  | **HA** | **NA** | **PB2** | **PB1** | **PA** | **NP** | **M** | **NS** |
| BD_11749 |  |  |  |  |  |  |  |  |
| BD_11758 |  |  |  |  |  |  |  |  |
| BD_11760 |  |  |  |  |  |  |  |  |
| BD_11787 |  |  |  |  |  |  |  |  |
| IN_3532 |  |  |  |  |  |  |  |  |
| IN_3533 |  |  |  |  |  |  |  |  |
| BD_3534 |  |  |  |  |  |  |  |  |
| IN_3535 |  |  |  |  |  |  |  |  |
| IN_117 |  |  |  |  |  |  |  |  |
| IN_118 |  |  |  |  |  |  |  |  |
| IN_119 |  |  |  |  |  |  |  |  |
| DU_120 |  |  |  |  |  |  |  |  |
| DU_121 |  |  |  |  |  |  |  |  |
| MO_166 |  |  |  |  |  |  |  |  |
| DE_142 |  |  |  |  |  |  |  |  |
| DE_143 |  |  |  |  |  |  |  |  |
| DE_144 |  |  |  |  |  |  |  |  |

Color codes: Green, H9N2-G1 lineage; Blue, H9 Korean lineage; Red, H5-clade 2.3.2.1; Brown, N8; Yellow, N3; Sky blue, European wild bird lineage
