# Supplementary material for: Comparison of pathogenicity of subtype H9 avian influenza wild-type viruses from a wide geographic origin expressing mono-, di-, or tri-basic hemagglutinin cleavage sites
Source: Vet Res. 2020 Mar 31;51:48. doi: 10.1186/s13567-020-00771-3 (PMC7106749; doi:10.1186/s13567-020-00771-3)
Supplement: Supplementary file 4 — Additional file 4. Analyses of substituting mutations described to increase pathogenicity and promoting mammalian adaptation of H9Nx viruses fully sequenced in this study. [file 13567_2020_771_MOESM4_ESM.docx]

**Additional file 4.** Analyses of substituting mutations described to increase pathogenicity and promoting mammalian adaptation of H9Nx viruses fully sequenced in this study.

| Strains/Isolates | **PB2**  [Gao et al., 2019; Xiao et al., 2016; Wang et al.,201652-54] | | | | **PB1-F2**  [Ozawa et al., 2011] | **NS1**  C-terminal  [ Ozawa et al., 2011] | **M1**  [Pu et al., 2017] | |
| --- | --- | --- | --- | --- | --- | --- | --- | --- |
|  | I292**V** | R340**K** | A588**V** | Q591**K** | N66**S** | 227-230 | V37**A** | R95**K** |
| BD_11749 | I | K | A | Q | N | KPEV | A | K |
| BD_11758 | V | R | A | Q | N | KSEV (1918-type) | A | K |
| BD_11760 | V | R | A | Q | N | KSEV | A | K |
| BD_11787 | V | R | A | Q | S | KSEV | A | K |
| IN_3532 | I | R | V | Q | N | KSEI | V | K |
| IN_3533 | I | R | V | Q | N | KSEI | V | K |
| BD_3534 | I | R | A | Q | N | KSEV (1918-type) | V | K |
| IN_3535 | I | R | V | Q | N | KSEV | V | K |
| IN_117 | I | R | V | Q | N | KSEV | V | K |
| IN_118 | I | R | V | Q | N | KSEI | V | K |
| IN_119 | I | R | V | Q | N | KSEI | V | K |
| DU_120 | I | K | A | K | N | GSEV | A | K |
| DU_121 | I | K | A | Q | N | GSEV | A | K |
| MO_166 | I | K | A | Q | N | GSEV | A | K |
| DE_142 | I | R | A | Q | N | ESEV (H5N1-type) | T | R |
| DE_143 | I | R | A | Q | N | ESEV | T | R |
| DE_144 | I | R | A | Q | N | ESEV | T | R |

Mutations are indicated red in color.

**Citations:**

Gao W, Zu Z, Liu J, Song J, Wang X, Wang C, Liu Tong Q, Wang M, Sun H, Sun Y, Liu J, Chang KC, Pu J (2019). Prevailing I292V PB2 mutation in avian influenza H9N2 virus increases viral polymerase function and attenuates IFN-beta induction in human cells. J Gen Virol 100:1273-1281

Xiao C, Ma W, Sun N, Huang L, Li Y, Zeng Z, Wen Y, Zhang Z, Li H, Li Q, Yu Y, Zheng Y, Liu S, Hu P, Zhang X, Ning Z, Qi W, Liao M (2016). PB2-588 V promotes the mammalian adaptation of H10N8, H7N9 and H9N2 avian influenza viruses. Sci Rep 6:19474

Wang C, Lee HH, Yang ZF, Mok CK, Zhang Z (2016). PB2-Q591K Mutation Determines the Pathogenicity of Avian H9N2 Influenza Viruses for Mammalian Species. PLoS One 11:e0162163.

Ozawa M, Basnet S, Burley LM, Neumann G, Hatta M, Kawaoka Y (2011). Impact of amino acid mutations in PB2, PB1-F2, and NS1 on the replication and pathogenicity of pandemic (H1N1) 2009 influenza viruses. J Virol 85:4596-4601

Pu J, Sun H, Qu Y, Wang C, Gao W, Zhu J, Sun Y, Bi Y, Huang Y, Chang KC, Cui J, Liu J. (2017). M Gene Reassortment in H9N2 Influenza Virus Promotes Early Infection and Replication: Contribution to Rising Virus Prevalence in Chickens in China. J Virol 91: e02055-16
