# Supplementary material for: Comparison of pathogenicity of subtype H9 avian influenza wild-type viruses from a wide geographic origin expressing mono-, di-, or tri-basic hemagglutinin cleavage sites
Source: Vet Res. 2020 Mar 31;51:48. doi: 10.1186/s13567-020-00771-3 (PMC7106749; doi:10.1186/s13567-020-00771-3)
Supplement: Supplementary file 5 — Additional file 5. Distribution in tissues of matrix protein immunoreactivity in mock and H9Nx infected chicken embryos assessed by semiquantitative scoring (0 = negative; 1 = focal/oligofocal; 2 = multifocal; 3 = coalescing/diffuse) and expressed as median values (M) with upper limit (UL) respectively lower limit (LL). [file 13567_2020_771_MOESM5_ESM.docx]

**Additional file 5.** Distribution in tissues of matrix protein immunoreactivity in mock and H9Nx infected chicken embryos assessed by semiquantitative scoring (0 = negative; 1 = focal/oligofocal; 2 = multifocal; 3 = coalescing/diffuse).

| **Tissues** | **H9 virus isolate including HACS sequence motif -P6 to –P1** | | | | | | | | | |
| --- | --- | --- | --- | --- | --- | --- | --- | --- | --- | --- |
|  | **Mock**  **(*n* = 2)^1^** | **BD_11749**  **PAKSKR tribasic**  **(*n* = 3)** | **IN_117**  **PAKSKR tribasic**  **(*n* = 2)** | **IN_118**  **PARSSR dibasic-1**  **(*n* = 2)** | **EG_536**  **PARSSR dibasic-1**  **(*n* = 2)** | **BD_VP01**  **PAKSSR dibasic-2**  **(*n* = 3)** | **MO_166**  **HARSSR dibasic-4**  **(*n* = 2)** | **DU_121**  **HARSSR, dibasic-4**  **(*n* = 2)** | **DE_234**  **PAASNR monobasic-1**  **(*n* = 2)** | **DE_142**  **PAASSR monobasic-2**  **(*n* = 2)** |
| chorionic membrane | 0 | 0 | 0 | 0 | 0 | 0 | 0 | 0 | 0 | 0 |
| allantoic membrane | 0 | **2** | **2** | **2** | **2** | **2** | **2** | **2** | **2** | **2** |
| nasal cavity | 0 | **3** | **3** | **3** | **2** | **2** | 0 | 0 | 0 | 0 |
| oral cavity | 0 | **2** | **2** | **2** | **3** | **2** | 0 | 0 | 0 | 0 |
| lung | 0 | **3** | **3** | **3** | **3** | 0 | 0 | 0 | 0 | 0 |
| air sac | 0 | **3** | **3** | **1** | **1** | 0 | 0 | 0 | 0 | 0 |
| heart | 0 | 0 | 0 | 0 | 0 | 0 | 0 | 0 | 0 | 0 |
| proventriculus | 0 | **2** | **2** | 0 | 0 | 0 | 0 | 0 | 0 | 0 |
| gizzard | 0 | **2** | **2** | 0 | 0 | 0 | 0 | 0 | 0 | 0 |
| intestine | 0 | 0 | 0 | **2** | **2** | 0 | 0 | 0 | 0 | 0 |
| pancreas | 0 | 0 | 0 | 0 | 0 | N/D | 0 | 0 | 0 | 0 |
| liver | 0 | 0 | 0 | 0 | 0 | 0 | 0 | 0 | 0 | 0 |
| kidney | 0 | 0 | 0 | **2** | **2** | 0 | 0 | 0 | 0 | 0 |
| gonad | 0 | 0 | 0 | 0 | 0 | 0 | 0 | 0 | 0 | 0 |
| spleen | 0 | 0 | 0 | 0 | 0 | 0 | 0 | 0 | N/D | N/D |
| skin | 0 | **3** | **3** | **2** | **2** | **2** | 0 | 0 | 0 | 0 |
| brain | 0 | 0 | 0 | 0 | 0 | 0 | 0 | 0 | 0 | 0 |
| **Σ^2^** | **0** | **20** | **20** | **17** | **17** | **8** | **2** | **2** | **2** | **2** |

^1^ Number of embryonated chicken eggs used for assessment.

^2^ Summed score of maximal values of matrixprotein immunoreactivity.

Bold numbers are the maximum values given by at least one embryo for each virus.

N/D – not determined
