# Supplementary material for: Comparison of pathogenicity of subtype H9 avian influenza wild-type viruses from a wide geographic origin expressing mono-, di-, or tri-basic hemagglutinin cleavage sites
Source: Vet Res. 2020 Mar 31;51:48. doi: 10.1186/s13567-020-00771-3 (PMC7106749; doi:10.1186/s13567-020-00771-3)
Supplement: Supplementary file 6 — Additional file 6. Pathogenicity indices and seroconversion of ten chickens each after intravenous inoculation of H9Nx viruses. [file 13567_2020_771_MOESM6_ESM.docx]

**Additional file 6.** Pathogenicity indices and seroconversion of ten chickens each after intravenous inoculation of H9Nx viruses.

| **Viruses** | **Subtype** | **Deduced HACS sequences** | | **Intravenous pathogenicity**  **index (IVPI)** | **Seroconversion^1^** | |
| --- | --- | --- | --- | --- | --- | --- |
|  |  | **Motif -P6 to -P1** | **Motif Type** |  | **NP_ELISA**  **bird/total** | **HI titer range** |
| BD_11749 | H9N2 | PAKSKR | Tribasic | 0 | 9/10 | 32-256 |
| IN_118 | H9N2 | PARSSR | Dibasic-1 | 0 | 8/10 | 32-128 |
| BD_VP01 | H9N2 | PAKSSR | Dibasic-2 | 0 | 9/10 | 16-128 |
| M0_166 | H9N2 | HARSSR | Dibasic-4 | 0 | 9/10 | 16-128 |
| DE_142 | H9N8 | PAASSR | Monobasic-2 | 0 | 4/10 | 0-8 |

^1^ Measured by NP blocking ELISA and hemagglutination inhibition (HI) test against the homologous antigen.

Red color highlights basic amino acids
